# Macrophage-linked lipid metabolic signatures for HCC prognosis identified through integrated bulk and single-cell transcriptomics

**DOI:** 10.3389/fimmu.2026.1898124

**Published:** 2026-09-03

**Authors:** Yu Yang, Manman Lu, Huapeng Zhang, Jinfu Zhang, Jinghua Yang, Shuai Xue

**Affiliations:** 1 Department of Hepatopancreatobiliary Surgery, The First Affiliated Hospital of Zhengzhou University, Zhengzhou, China; 2 Center for Reproductive Medicine, The First Affiliated Hospital of Zhengzhou University, Zhengzhou, Henan, China; 3 Clinical Systems Biology Laboratories, Translational Medicine Center, The First Affiliated Hospital of Zhengzhou University, Zhengzhou, China

**Keywords:** hepatocellular carcinoma, lipid metabolism, macrophages, prognosis, single-cell RNA sequencing

## Abstract

**Background:**

Macrophage-dependent immune remodeling and altered lipid metabolism are closely related to liver cancer behavior. In hepatocellular carcinoma (HCC), the survival relevance of macrophage-associated lipid metabolism genes (MLMGs) remains insufficiently characterized. This work evaluated MLMG-related molecular features connected with HCC progression and prognosis.

**Methods:**

Public HCC transcriptomic and single-cell cohorts were analyzed. Macrophage-related differential genes (MRDGs) extracted from GSE149614 were combined with TCGA-HCC differentially expressed genes1 (DEGs1) and lipid metabolism-related genes (LMRGs). Shared candidates were screened through univariate Cox analysis and machine-learning procedures to obtain prognostic MLMGs and build a risk-score model. Immune profiling, enrichment analysis, cell-cell communication inference, pseudo-time reconstruction, and RT-qPCR validation were then performed.

**Results:**

FABP5 and ECHS1 constituted the final two-gene prognostic signature. Risk categories were accompanied by altered immune components, including activated B cells, and pathways such as “fatty acid omega hydroxylase activity”. The high-risk group suggested potentially stronger immune escape-related features and higher checkpoint expression. Communication analysis suggested stronger interactions between CD8+ T cells and natural killer (NK) cells, whereas pseudotime analysis showed that MLMG expression may be associated with changes in CD8+ T-cell states. RT-qPCR preliminarily validated increased FABP5 expression and decreased ECHS1 expression in patients with HCC.

**Conclusion:**

FABP5 and ECHS1 were selected as prognostic MLMGs in HCC. This two-gene model may provide clues for survival assessment and subsequent mechanism-oriented research.

## 1 Introduction

HCC is a leading malignant disease worldwide, ranking sixth for incidence and third for cancer-related mortality. Even after surgery, relapse affects approximately 40% to 50% of patients within 2 years and 60% to 70% within 5 years (1, 2). Early diagnosis remains difficult because ultrasound is less reliable in obese or steatotic patients, alpha-fetoprotein identifies only about 49% of early-stage tumors, and biopsy is less successful for nodules below 2 cm. More than half of Chinese patients are therefore diagnosed after curative resection is no longer optimal (3, 4). Existing therapies also have clear restrictions: thermal ablation performs poorly for tumors >3 cm or those near major vessels, transarterial chemoembolization produces complete response in only 14% of tumors beyond the Up-to-7 criteria, and transplantation depends on organ availability and the Milan criteria (5). First-line immunotherapy combinations have improved treatment, but only about 20% to 40% of patients respond objectively, and resistance often develops within 4 to 8 months under immunosuppressive pressure (6). These limitations make robust prognostic markers and actionable targets necessary.

Within the HCC microenvironment, tumor-associated macrophages (TAMs) are highly abundant and phenotypically flexible. They range from anti-tumorigenic M1-like cells to pro-tumorigenic M2-like cells, and M2-polarized TAM accumulation is closely linked to metastasis and poor prognosis (7). Cytokines and exosomes from M2 TAMs activate NF-κB, Smad, and Wnt/β-catenin signaling, induce EMT and stemness, enhance VEGF- and CCR2-dependent angiogenesis, inhibit CD8^+^ T-cell cytotoxicity through suppressive ligands, and recruit regulatory T cells, jointly establishing an immunosuppressive niche (8, 9). Lipid metabolic disturbance, commonly associated with obesity, diabetes, and NASH, is another hallmark of HCC (10). Rewired lipid metabolism supplies energy and membrane materials, changes immune-cell behavior, and promotes therapy resistance (11). HCC cells frequently increase fatty acid uptake and lipogenic enzyme activity, while fatty acid β-oxidation and cholesterol synthesis are remodeled (12, 13). Lipid-derived signals can further activate PI3K/AKT and Wnt/β-catenin pathways, driving M2-like TAM polarization, Treg recruitment, disease progression, and treatment failure (14). These overlapping immune and metabolic changes provide a rationale for studying MLMGs in HCC.

scRNA-seq captures transcriptomes at single-cell resolution and avoids the averaging effect of bulk sequencing. By retaining rare cell states, intercellular heterogeneity, and dynamic trajectories, it is well suited for examining immune-metabolic interactions (15). In HCC, single-cell analyses have described exhausted CD8^+^ T cells, TAMs, CD36+ cancer-associated fibroblasts, SPP1-CD44-mediated immune escape, lipid-metabolism gene remodeling, and ligand-receptor crosstalk such as MIF-CD74 and CD86-CTLA4 (16, 17). These features suggest that scRNA-seq can improve prognostic MLMG discovery and assist patient stratification and treatment-response monitoring.

Here, transcriptomic and scRNA-seq datasets were integrated to investigate macrophage-related lipid metabolic reprogramming in HCC. We established an MLMG-based prognostic model, validated it externally, and compared immune features, pathway activity, and mutation patterns between risk groups. The findings suggest that MLMGs have potential prognostic significance and may provide clues for HCC risk assessment and target selection.

## 2 Materials and methods

### 2.1 Data acquisition

RNA-seq profiles, clinical annotations, and survival information for HCC were downloaded from the TCGA database on December 29, 2024. The TCGA-HCC training cohort comprised 374 HCC tumor tissue samples (HCC group) and 50 normal controls (normal group), including 365 HCC tumor samples with survival data. The HCC validation set GSE14520 and the scRNA-seq dataset GSE149614 were obtained from GEO. GSE14520 (platform: GPL3921) contained 225 HCC tumor tissue samples and 220 normal controls; 215 HCC samples with survival information were retained for risk-model validation. GSE149614 (platform: GPL24676) included 10 primary HCC samples and 8 normal controls. A total of 743 LMRGs (**Table 1**) were retrieved from MSigDB (18). All datasets were used in accordance with their database access policies.

**Table 1:** The primer sequences.

Primer	Sequence
ECHS1-F	TGCCACTGATGACCGGAAAG
ECHS1-R	GCACTTGTCCTCTCCAAGCA
FABP5-F	AGGAGTGGGAATAGCTTTGCG
FABP5-R	GCTGAACCAATGCACCATCT
GAPDH-F	ATGGGCAGCCGTTAGGAAAG
GAPDH-R	AGGAAAAGCATCACCCGGAG

### 2.2 Identification of MRDGs using scRNA-seq data

The Seurat package (v 5.1.0) was used to extract MRDGs from GSE149614 (19). Cells were retained when nFeature_RNA ranged from 250 to 6,000, mitochondrial proportion was <20%, and cluster size was at least 3 cells. The matrix was normalized with NormalizeData, and the top 2,000 highly variable genes were selected by FindVariableFeatures. PCA was conducted with RunPCA and JackStraw, and significant PCs were kept (p < 0.05). Unsupervised clustering and visualization used t-SNE through RunTSNE, FindNeighbors, and FindClusters (resolution = 1.0). Cell identities were assigned according to marker genes identified by FindAllMarkers and the CellMarker database, and marker expression across annotated cells was shown with a bubble plot.

For every annotated cell type, abundance was compared between HCC and normal samples. Cell populations with significant abundance changes (p < 0.05) were considered differential cells. ReactomeGSA (v 1.14.0) was used to explore pathways associated with annotated cells during HCC development (p < 0.05) (20). Spearman correlations among pathway expression profiles were summarized with the psych package (v 2.4.6.26) as a heatmap (|cor| > 0.30, p < 0.05) (21). MRDGs were then identified in differential macrophages between HCC and normal samples using FindMarkers (|average log_2_ FC| > 0.25, adj.p < 0.05).

### 2.3 Functional enrichment analysis of DE-MLMGs

DEGs1 in TCGA-HCC were determined by comparing HCC and normal samples with DESeq2 (v 1.40.2) (|log_2_ FC| > 1, adj.p < 0.05). Volcano and heatmap figures were drawn using ggplot2 (v 3.5.1) and ComplexHeatmap (v 2.16.0), respectively (22–24). UpSetR (v 1.4.0) was applied to intersect MRDGs, LMRGs, and DEGs1, yielding DE-MLMGs (25). Their biological implications were assessed by GO and KEGG enrichment analyses with clusterProfiler (v 4.15.0.003) (adj.p < 0.05) (26). A PPI network was further generated in the STRING database (interaction score > 0.4) to examine relationships among DE-MLMG-encoded proteins.

### 2.4 Construction and verification of risk model

In TCGA-HCC samples with survival data, candidate prognostic MLMGs were selected from DE-MLMGs using univariate Cox regression analysis (HR = 1, p < 0.05) plus PH assumption testing (p > 0.05) in the survival package (v 3.7-0) (27). Model refinement used LASSO regression implemented in glmnet (v 4.1-8) with 10-fold cross-validation (28). The selected genes were incorporated into the risk-score formula:


$$risk\;core=\sum_{i=1}^{N}\text{coef}({\text{gene}}_{i})\times \text{expr}({\text{gene}}_{i})$$

Here, coef indicates the risk coefficient, expr indicates the expression value of each prognostic MLMG, and N indicates the total number of prognostic MLMGs in the model. According to the optimal risk-score cutoff, TCGA-HCC patients were assigned to the HRG or LRG. Risk-score distribution, survival status, and prognostic MLMG expression were visualized. K-M curves were generated with the survival package (v 3.7-0) (log-rank test, p < 0.05), and ROC curves were generated with timeROC (v 0.4) (29). AUC values ≥0.6 for 1-, 2-, and 3-year survival were interpreted as moderate discrimination. The GSE14520 cohort was used to assess external robustness and predictive accuracy.

### 2.5 Independent prognostic analysis

In TCGA-HCC cases with survival information, the prognostic independence of the Risk Score and conventional clinical characteristics was evaluated. The clinical variables included age, sex, tumor stage, TNM stage, and Risk Score. Univariate and multivariate Cox regression analyses were performed using the survival package (v 3.7-0) (HR ≠ 1, p < 0.05), and the proportional hazards assumption was assessed using Schoenfeld residual tests (p > 0.05).

Considering that the liver fibrosis/cirrhosis background may affect the prognosis of patients with HCC, we further included the Ishak fibrosis score in addition to the original clinical variables for supplementary analysis. Because Ishak fibrosis score data were missing in the TCGA-HCC cohort, only cases with complete Ishak fibrosis score, conventional clinical information, and survival information were retained for complete-case analysis. First, univariate Cox regression was used to evaluate the associations between each variable and overall survival, and Schoenfeld residual tests were performed. Subsequently, variables that met the proportional hazards assumption and were statistically significant were included in a multivariate Cox regression model to evaluate the prognostic robustness of the Risk Score after adding the Ishak fibrosis score. The car package was further used to calculate the variance inflation factor (VIF) of variables in the multivariate model to assess multicollinearity; VIF < 5 was considered to indicate no obvious multicollinearity. A nomogram was constructed based on the independent prognostic factors determined by the supplementary multivariate Cox analysis, and calibration curves and decision curve analysis were used to evaluate its predictive agreement and potential clinical net benefit.

### 2.6 Immune microenvironment analysis

Because immune heterogeneity in the tumor microenvironment is strongly associated with HCC progression and treatment resistance (30), immune infiltration was assessed in TCGA-HCC. The GSVA package (v 1.53.28) and GSEABase package (v 1.67.1) were used to run ssGSEA, which calculated infiltration scores for 28 immune infiltrated cells and quantified immune-related pathway activity between the HRG and LRG (31–33). Differential immune cells and pathways were identified by the Wilcoxon test (p < 0.05).

Immune score, stromal score, ESTIMATE score, and tumor purity for both risk groups were obtained with the estimate package (v 1.0.13) and compared using the Wilcoxon test (p < 0.05) (34). Checkpoint expression was also compared between groups because tumor cells can use these molecules to weaken immune-mediated killing (Wilcoxon test, p < 0.05) (35). Spearman correlations among prognostic MLMGs, risk score, differential immune cells, differential immune-related pathways, immune scores, stromal scores, ESTIMATE scores, tumor purity, and differential immune checkpoints were calculated with psych (v 2.4.6.26) (|cor| > 0.30, p < 0.05).

To further evaluate the external reproducibility of immune features associated with risk stratification, the Risk Score for each patient in the GSE14520 validation cohort was calculated using the same risk-score formula, and patients were divided into high- and low-risk groups according to the grouping method determined in the training cohort. Subsequently, ssGSEA was used to evaluate immune-cell infiltration and immune-related functional scores, and the ESTIMATE algorithm was used to calculate the ESTIMATE score, ImmuneScore, StromalScore, and TumorPurity. Differences in immune checkpoint gene expression between the high- and low-risk groups were further compared. Between-group differences were assessed using the Wilcoxon rank-sum test, and p < 0.05 was considered statistically significant.

To explore the relationship between the Risk Score and potential immunotherapy response, the TIDE algorithm was further used to evaluate immune escape-related scores in patients from the high- and low-risk groups, including the TIDE score, cell dysfunction score, and cell exclusion score. Differences in TIDE-related scores between risk groups were then compared to preliminarily assess the potential relationship between the Risk Score and immune escape-related features.

### 2.7 GSEA

Functional differences between the HRG and LRG in TCGA-HCC were examined by GSEA. Differential expression analysis between the 2 risk groups was first carried out with DESeq2 (v 1.40.2). The log_2_FC values of DEGs2 were ranked in ascending order and analyzed using clusterProfiler (v 4.15.0.003) under FDR < 0.05 and p < 0.05. Background gene sets, including “c2.cp.kegg.v7.5.symbols” and “c5.kegg.v7.4.symbols.gmt”, were downloaded from MSigDB.

### 2.8 Correlation analysis between mechanism-related pathway scores and prognostic MLMGs

To further evaluate the relationship between prognostic MLMGs and mechanism-related pathway activity, gene sets related to PI3K, AKT, autophagy, and ferroptosis were downloaded from the GSEA database. Based on the TCGA-HCC expression matrix, ssGSEA in the GSVA package was used to calculate pathway activity scores for each sample. Subsequently, Spearman correlation analysis was used to evaluate the correlations between prognostic gene expression levels and ssGSEA scores of each pathway. The correlation results were displayed as a heatmap, and p < 0.05 was considered statistically significant.

### 2.9 Genetic mutation analysis and epigenetic analysis

Genetic mutation analysis quantifies non-synonymous mutations per megabyte and reflects genomic instability in HCC (36). For TCGA-HCC, somatic mutation data (Masked Somatic Mutation, MAF format) and CNV data (Masked Copy Number Segment) for the HRG and LRG were downloaded from the GDC Data Portal. Somatic mutations were analyzed in R using maftools (v2.18.0). CNV segment files were processed by GISTIC2.0 (v2.0.23) to identify significant amplification or deletion regions (q < 0.25). To further evaluate the relationship between mutation status and the risk score, TCGA-HCC patients were divided into mutant (MT) and wild-type (WT) groups based on the mutation status of each key gene identified from the mutational landscape analysis. Subsequently, differences in risk scores between patients with different mutation statuses were compared, and statistical differences were calculated using the Wilcoxon rank-sum test. Violin plots were used to display the distribution of risk scores among different mutation status groups, and p < 0.05 was considered statistically significant.

Because epigenetic regulation, including chromatin remodeling, histone modification, DNA methylation, and non-coding RNA (ncRNA) expression, contributes to HCC progression and metastasis (37), CPTAC data were used to compare total protein levels of prognostic MLMGs between normal and HCC samples (Wilcoxon test, p < 0.05). DNA methylation sites were assessed in UALCAN (Wilcoxon test, p < 0.05).

### 2.10 Analysis of cell communication, pseudotime, and subgroup expression of key cells and macrophages

All GSE149614 samples were used for key-cell selection. Differential cells were selected when they expressed prognostic MLMGs, showed significant expression differences between normal and HCC samples (p < 0.05), and were significantly associated with HCC progression. CellChat (v 1.6.1) was used to infer cell-cell communication among annotated cells (38), and a bubble plot displayed receptor-ligand interactions between key cells and other annotated populations. Key cells were then subjected to secondary dimensionality reduction and clustering (resolution = 0.4) following Section 2.2 and were annotated into subgroups. Pseudo-time analysis was performed with monocle (v 2.28.0) (39), and prognostic MLMG expression was evaluated along key-cell differentiation.

To further evaluate the relationship between prognostic MLMGs and different subpopulation states of key cells, we extracted the expression matrix of prognostic genes in each subpopulation based on the above key-cell subpopulation annotations. Subsequently, within each key-cell subpopulation, the expression differences of prognostic genes between the HCC and normal groups were compared, and statistical significance was calculated using the Wilcoxon rank-sum test. The results were presented as violin plots (p < 0.05).

Considering that the model was derived from macrophage-related lipid metabolism genes, to further evaluate the expression characteristics of prognostic genes during changes in macrophage states, we extracted M1 and M2 macrophages from the GSE149614 single-cell dataset and combined them as a macrophage population for supplementary analysis. First, macrophages were reclustered using the FindNeighbors and FindClusters functions in the Seurat package, with the resolution set to 0.4, and were visualized by t-SNE. Subsequently, pseudotime trajectory analysis of macrophages was performed using the monocle package to infer the process of macrophage state transition. Furthermore, based on the M1 and M2 macrophage annotation results, the expression differences of prognostic genes between the HCC and normal groups were compared in M1 and M2 macrophages, respectively, and statistical significance was assessed using the Wilcoxon rank-sum test.

### 2.11 Expression levels of prognostic MLMGs

Prognostic MLMG expression in TCGA-HCC was compared between HCC and normal groups by the Wilcoxon test (p < 0.05).

### 2.12 RT-qPCR

Twenty HCC patient tissues and twenty control samples were collected from The First Affiliated Hospital of Zhengzhou University. All participants provided written informed consent, and ethical approval was granted by 2023-KY-1167-002. Detailed clinical information of the participants is provided in **Supplementary Table 2**. Total RNA was isolated using Trizol reagent (Ambion, Texas, USA). cDNA synthesis was performed with the SweScript First Strand cDNA synthesis kit (Servicebio, Wuhan, China). Amplification followed the manufacturer’s instructions, and PCR was run in a 10 μL reaction system. Relative mRNA levels of prognostic MLMGs were calculated by the 2^−ΔΔCt^ method. RT-qPCR primer pairs were designed by Sangon, Shanghai, and sequences are listed in **Table 1**. GAPDH served as the normalization reference.

### 2.13 Statistical analysis

R software (v 4.3.2) and GraphPad Prism (v 10) were used for statistical analyses. Relative mRNA expression levels of prognostic MLMGs were compared by t-test. Other group comparisons used the Wilcoxon test, and p < 0.05 was regarded as statistically significant.

## 3 Results

### 3.1 Identification of 1,212 MRDGs in comprehensive HCC single-cell landscape

**Figure 1:**
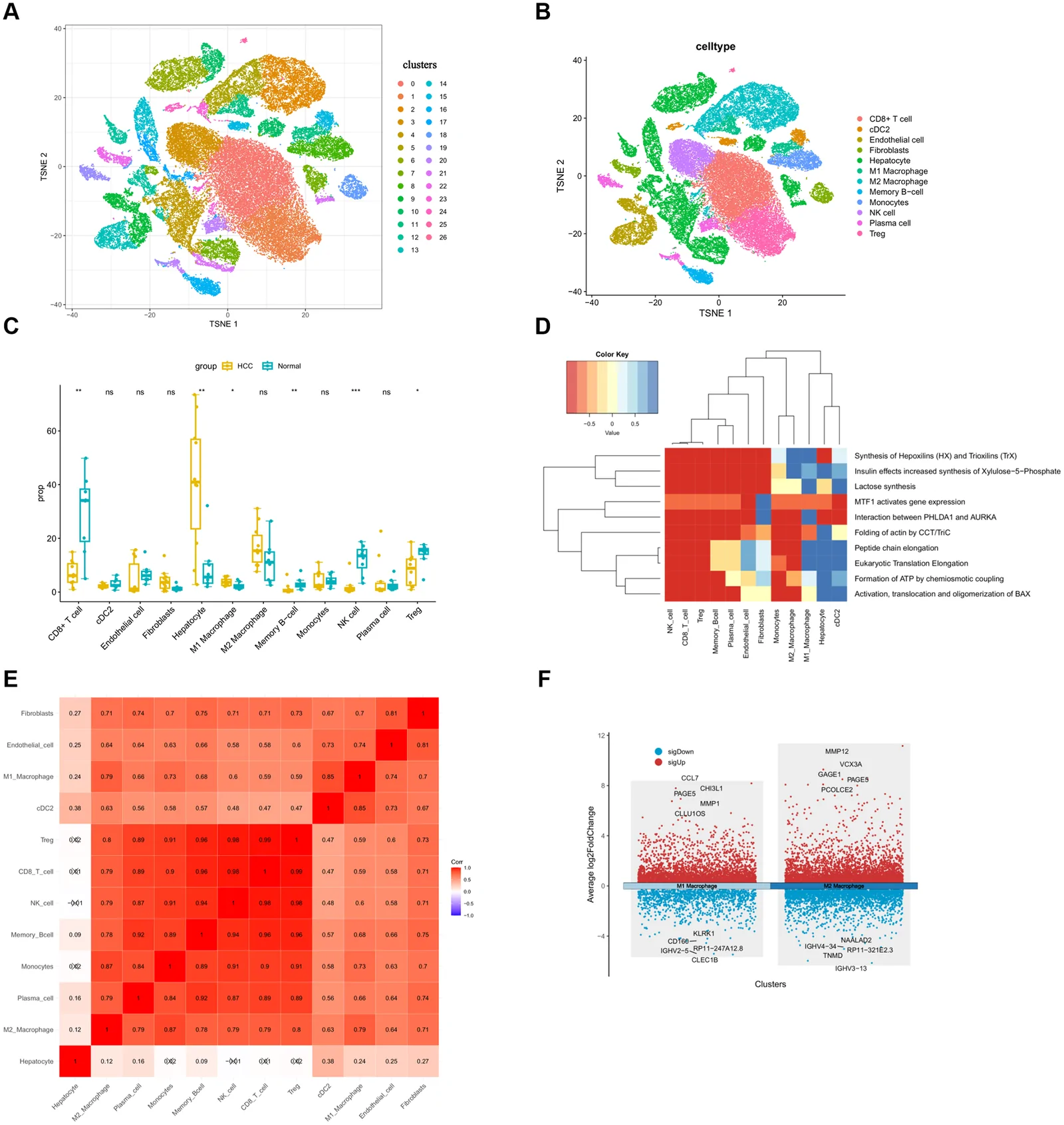
Cell clustering, annotation, differential cell types, pathway enrichment, and identification of MRDGs in the comprehensive HCC single-cell landscape. **(A)** t-SNE plot showing 27 cell clusters. **(B)** Cell type annotation: 12 cell types. **(C)** Cell types with significant abundance differences between HCC and normal samples (CD8+ T cells, hepatocytes, M1 macrophages, memory B cells, NK cells, Tregs; p < 0.05). **(D)** Significant pathways enriched in the annotated cells. **(E)** Spearman correlation heatmap of pathway expression levels; the strongest correlation was observed between CD8+ T cells and Tregs (cor = 0.99, p < 0.0001). **(F)** Differential expression analysis in M1 macrophages identified 1,212 MRDGs (881 up-regulated, 331 down-regulated).

After integration and filtering of GSE149614, the matrix contained 63,101 cells and 25,712 genes before QC, while 61,459 cells and 25,479 genes remained after QC (**Supplementary Figure 1A**). The top 2,000 highly variable genes were selected (**Supplementary Figure 1B**). After normalization, PCA and the scree plot supported retention of the top 20 PCs (p < 0.05, **Supplementary Figures 1C, D**). t-SNE separated the cells into 27 clusters (**Figure 1A**). These clusters were annotated as 12 cell types: CD8^+^ T cells, M1 macrophages, NK cells, M2 macrophages, endothelial cells, regulatory T cells (Tregs), plasma cells, monocytes, classical dendritic cells type 2 (cDC2), fibroblasts, hepatocytes, and memory B cells (**Figure 1B**). Marker-gene expression was visualized by bubble plot (**Supplementary Figure 1E**; **Supplementary Table 3**). CD8^+^ T cells, hepatocytes, M1 macrophages, memory B cells, NK cells, and Tregs showed significant abundance differences between HCC and normal samples (p < 0.05) and were defined as differential cells (**Figure 1C**).

The annotated cells were enriched in 1,975 significant pathways, including “MTF1 activates gene expression”, “folding of actin by CCT/TriC”, “peptide chain elongation”, “eukaryotic translation elongation”, and “lactose synthesis” (**Figure 1D**; **Supplementary Table 4**). A Spearman heatmap of pathway expression showed broad positive correlations among annotated cells; the strongest correlation occurred between CD8+ T cells and Tregs (cor = 0.99, p < 0.0001) (**Figure 1E**; **Supplementary Table 5**). In M1 macrophages, differential expression analysis produced 1,212 MRDGs, including 881 up-regulated and 331 down-regulated genes, for subsequent analyses (**Figure 1F**; **Supplementary Table 6**).

### 3.2 Identification and functional assessment of 20 DE-MLMGs

**Figure 2:**
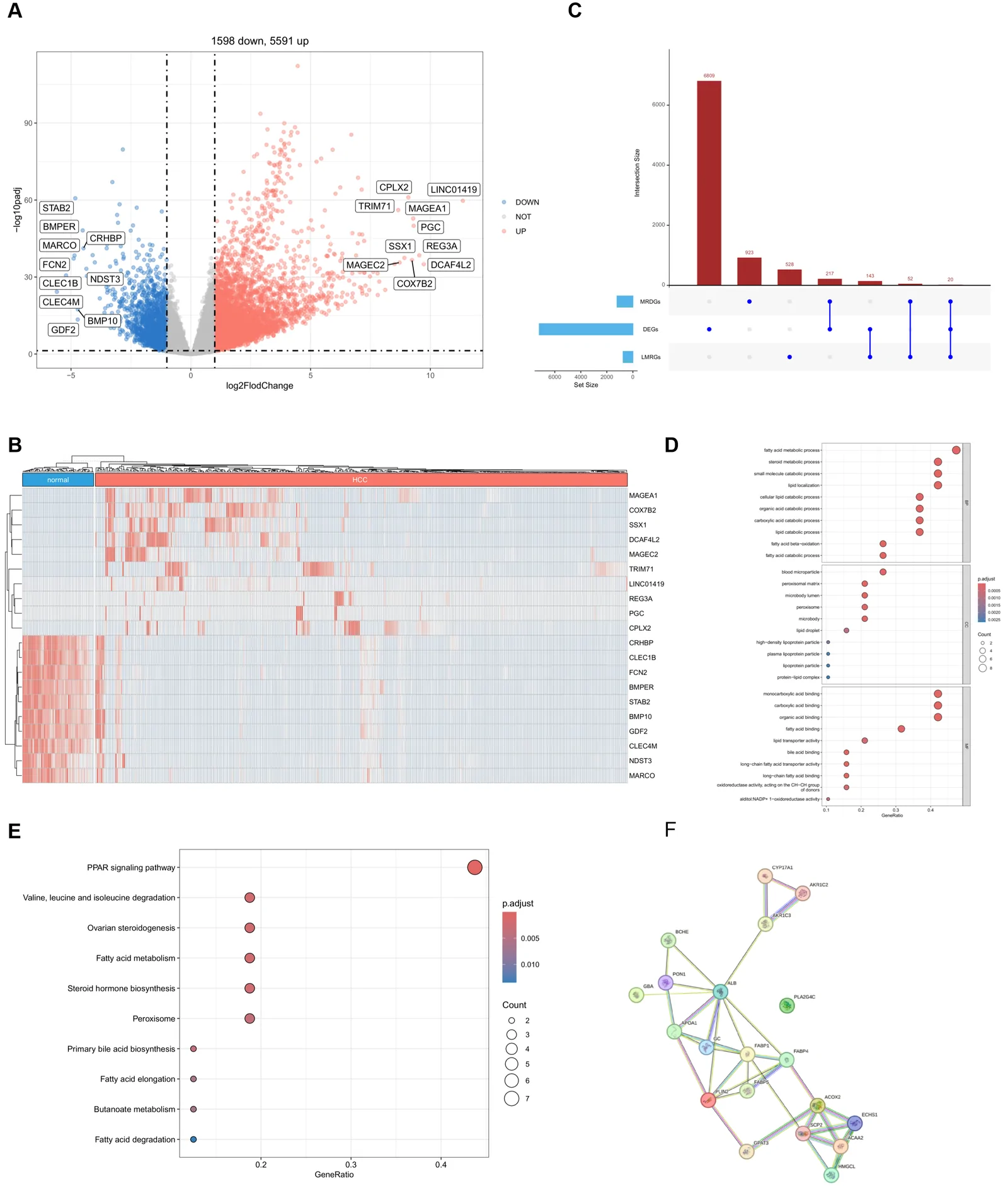
Identification, functional enrichment, and protein protein interaction network of DE MLMGs. **(A)** Volcano plot displaying the top 10 up and down regulated DEGs in TCGA HCC. **(B)** Heatmap showing distinct expression patterns of DEGs between HCC and normal groups. **(C)** Histogram intersecting 7,189 DEGs1, 1,212 MRDGs, and 743 LMRGs, yielding 20 DE MLMGs. **(D-E)** GO and KEGG enrichment analysis results. **(F)** PPI network of the 20 DE MLMG encoded proteins, highlighting highly connected nodes.

TCGA-HCC analysis detected 7,189 DEGs1 (**Supplementary Table 7**), with 5,591 up-regulated and 1,598 down-regulated genes in HCC samples. The volcano plot listed the top 10 up- and down-regulated DEGs1 by descending log2 FC (**Figure 2A**), and the heatmap showed distinct expression patterns between HCC and normal groups (**Figure 2B**). Intersecting 7,189 DEGs1, 1,212 MRDGs, and 743 LMRGs produced 20 DE-MLMGs: ACAA2, ACOX2, AKR1C2, AKR1C3, ALB, APOA1, BCHE, CYP17A1, ECHS1, FABP1, FABP4, FABP5, GBA, GC, GPAT3, HMGCL, PLA2G4C, PLIN2, PON1, and SCP2 (**Figure 2C**).

GO and KEGG enrichment analyses were used to interpret the 20 DE-MLMGs. GO analysis identified 298 significant terms, including “fatty acid metabolic process”, “steroid metabolic process”, “peroxisomal matrix”, “microbody lumen”, “monocarboxylic acid binding”, and “fatty acid binding” (adj.p < 0.05, **Figure 2D**; **Supplementary Table 8**). KEGG analysis identified 12 pathways, including “PPAR signaling pathway”, “ovarian steroidogenesis”, “fatty acid metabolism”, “primary bile acid biosynthesis”, and “peroxisome” (adj.p < 0.05, **Figure 2E**; **Supplementary Table 9**). The PPI network contained 20 proteins and 74 interaction pairs. ALB, APOA1, GC, FABP1, FABP4, FABP5, PLIN2, ACOX2, and SCP2 were highly connected (**Figure 2F**), suggesting potential involvement in HCC-related molecular regulation.

### 3.3 Good prediction performance of prognostic MLMG-related risk model

**Figure 3:**
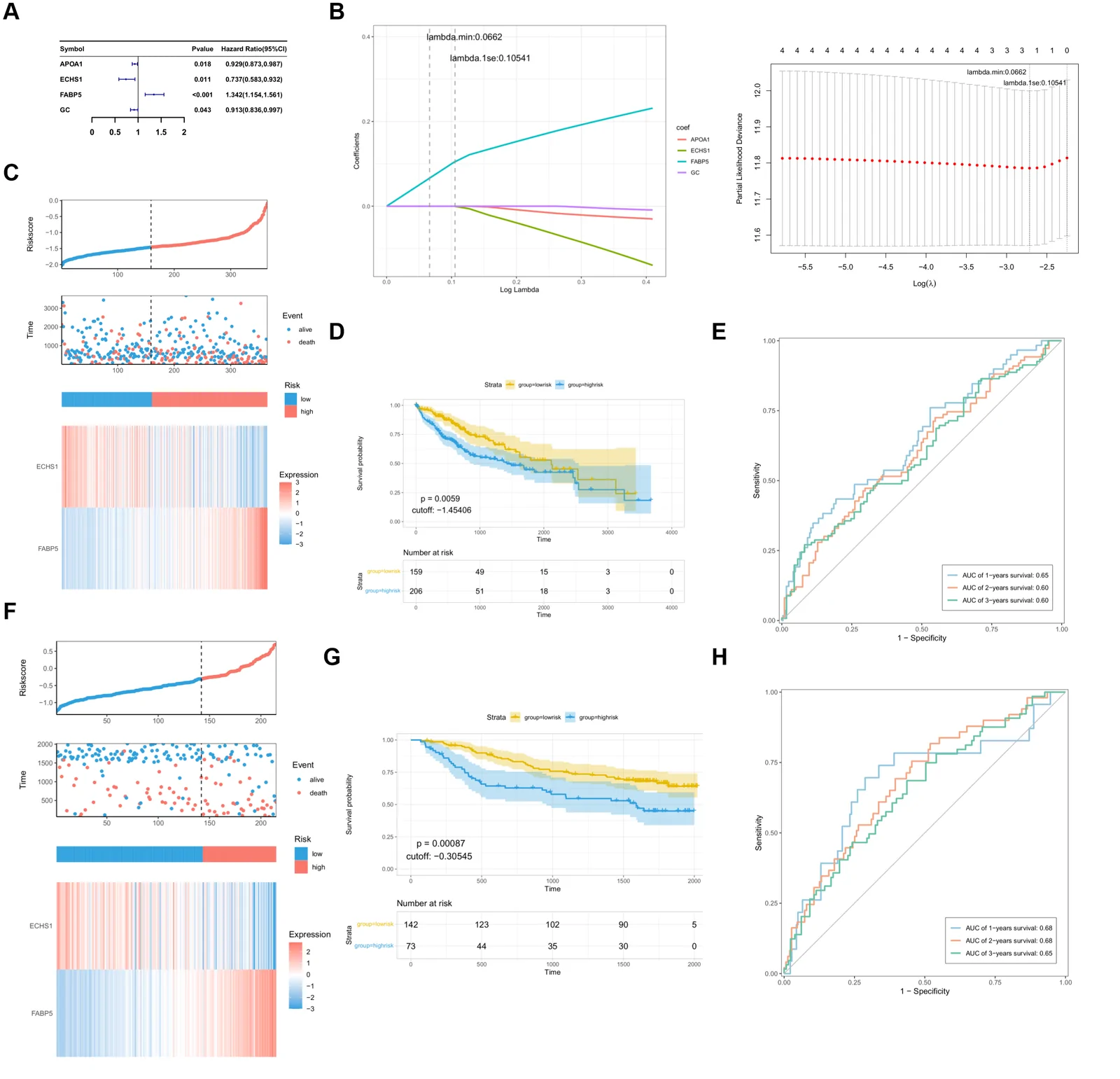
Construction and validation of a prognostic MLMG−based risk model. **(A)** Forest plot of univariate Cox regression showing FABP5 as a risk factor and ECHS1, APOA1, GC as protective factors. **(B)** LASSO regression cross−validation plot selecting FABP5 and ECHS1. **(C)** HRG and LRG groups in TCGA−HCC stratified by the optimal cutoff: upper panel, risk score distribution; middle panel, survival status; lower panel, expression heatmap of FABP5 and ECHS1. **(D)** Kaplan−Meier survival curve showing significantly poorer overall survival in the HRG. **(E)** Time−dependent AUC curves. **(F)** Risk score distribution, survival status, and expression heatmap in the validation set GSE14520. **(G)** Kaplan−Meier survival curve in the validation set GSE14520. **(H)** Time−dependent AUC curves in the validation set GSE14520.

Univariate Cox regression with PH assumption testing identified 4 candidate prognostic MLMGs associated with overall survival (OS) in TCGA-HCC. FABP5 behaved as a risk factor (HR > 1), whereas ECHS1, APOA1, and GC behaved as protective factors (HR < 1) (**Figure 3A**, **Supplementary Figure 2**). Ten-fold cross-validated LASSO regression (lambda.min = 0.0662) reduced these candidates to FABP5 and ECHS1 (**Figure 3B**). The risk formula was risk score = (-0.1982) * ECHS1 expression + (0.2526) * FABP5 expression. Using the optimal TCGA-HCC cutoff (-1.45406), 206 patients were assigned to the HRG and 159 to the LRG. The HRG showed more deaths and lower survival probability, with higher FABP5 and lower ECHS1 expression (**Figure 3C**). KM analysis confirmed poorer survival in high-risk patients (p = 0.0059, **Figure 3D**). The 1-, 2-, and 3-year OS AUC values were 0.65, 0.60, and 0.60 (**Figure 3E**). Similar results in GSE14520 supported validation-set stability (**Figures 3F–H**).

### 3.4 Risk score as independent prognostic factor in HCC

**Figure 4:**
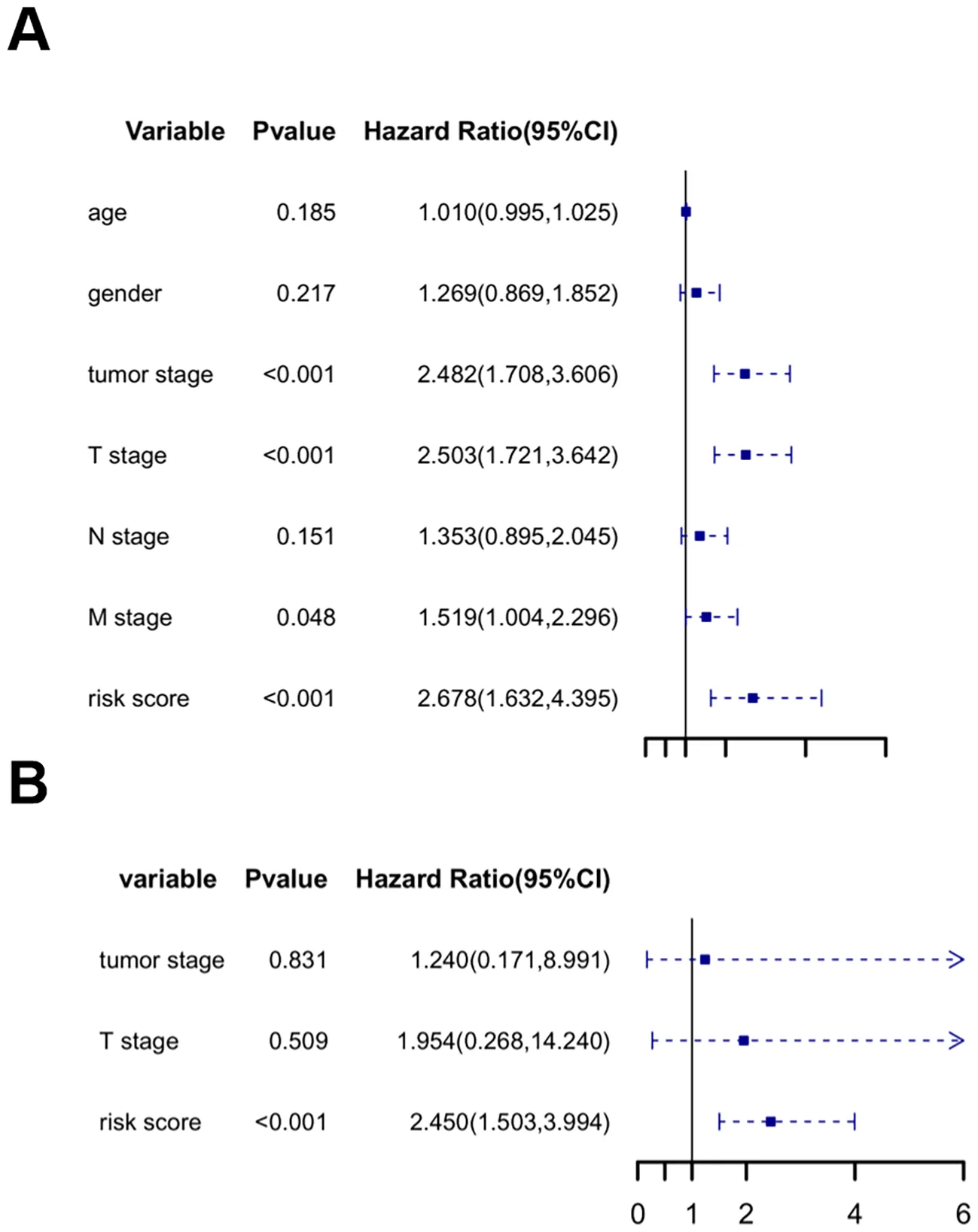
Independent prognostic value of the risk score in HCC. **(A)** Forest plot of univariate Cox regression showing tumor stage, T stage, and risk score as significant prognostic variables. **(B)** Forest plot of multivariate Cox regression demonstrating that risk score is independently associated with HCC prognosis.

Independent prognostic analysis evaluated the clinical value of the risk score. In TCGA-HCC, univariate Cox regression with PH assumption testing identified tumor stage, T stage, and risk score as significant prognostic variables (**Figure 4A**; **Supplementary Figure 3A**). Multivariate Cox regression with PH assumption testing further showed that risk score remained independently associated with HCC prognosis (**Figure 4B**; **Supplementary Figure 3B**). This suggested that the Risk Score may provide supplementary prognostic information beyond traditional clinicopathological features.

Considering that the liver fibrosis/cirrhosis background may affect the prognosis of patients with HCC, we further performed supplementary adjustment for clinical confounders in TCGA-HCC cases with complete Ishak fibrosis score information, including 181 patients. After age, sex, tumor stage, TNM stage, Ishak fibrosis score, and Risk Score were included in the analysis, univariate Cox regression showed that age, N stage, M stage, and Risk Score were significantly associated with OS; among them, the Risk Score still showed a strong prognostic association (HR = 4.213, 95% CI: 1.846-9.618, p = 0.001), whereas the Ishak fibrosis score showed no significant prognostic value (**Supplementary Figure 4A**). The PH assumption test showed that the Risk Score satisfied the proportional hazards assumption (p = 0.8163) (**Supplementary Figure 4B**). Further multivariate Cox regression analysis showed that age (HR = 1.055, 95% CI: 1.026-1.085, p < 0.001) and Risk Score (HR = 4.100, 95% CI: 1.822-9.224, p < 0.001) remained independent prognostic factors (**Supplementary Figure 4C**), and both satisfied the PH assumption (**Supplementary Figure 4D**). Collinearity assessment showed that the VIF values for age and Risk Score were both approximately 1.0008, suggesting no obvious multicollinearity in the multivariate model. The nomogram constructed based on age and Risk Score could be used to predict 1-, 2-, and 3-year OS in patients with HCC. Calibration curves showed a certain degree of agreement between predicted survival and observed outcomes, and DCA suggested that the model had potential clinical net benefit within some threshold ranges (**Supplementary Figures 4E–K**). Therefore, the supplementary analysis incorporating the Ishak fibrosis score controlled, to some extent, for the important clinical factor of liver fibrosis/cirrhosis background and further supported the independent prognostic value of the Risk Score in the complete-case cohort; however, because information on AFP level, viral infection status, and treatment regimens was frequently missing, its clinical robustness still needs to be validated in cohorts with more complete information.

### 3.5 Immune landscape associated with risk scores in HCC

**Figure 5:**
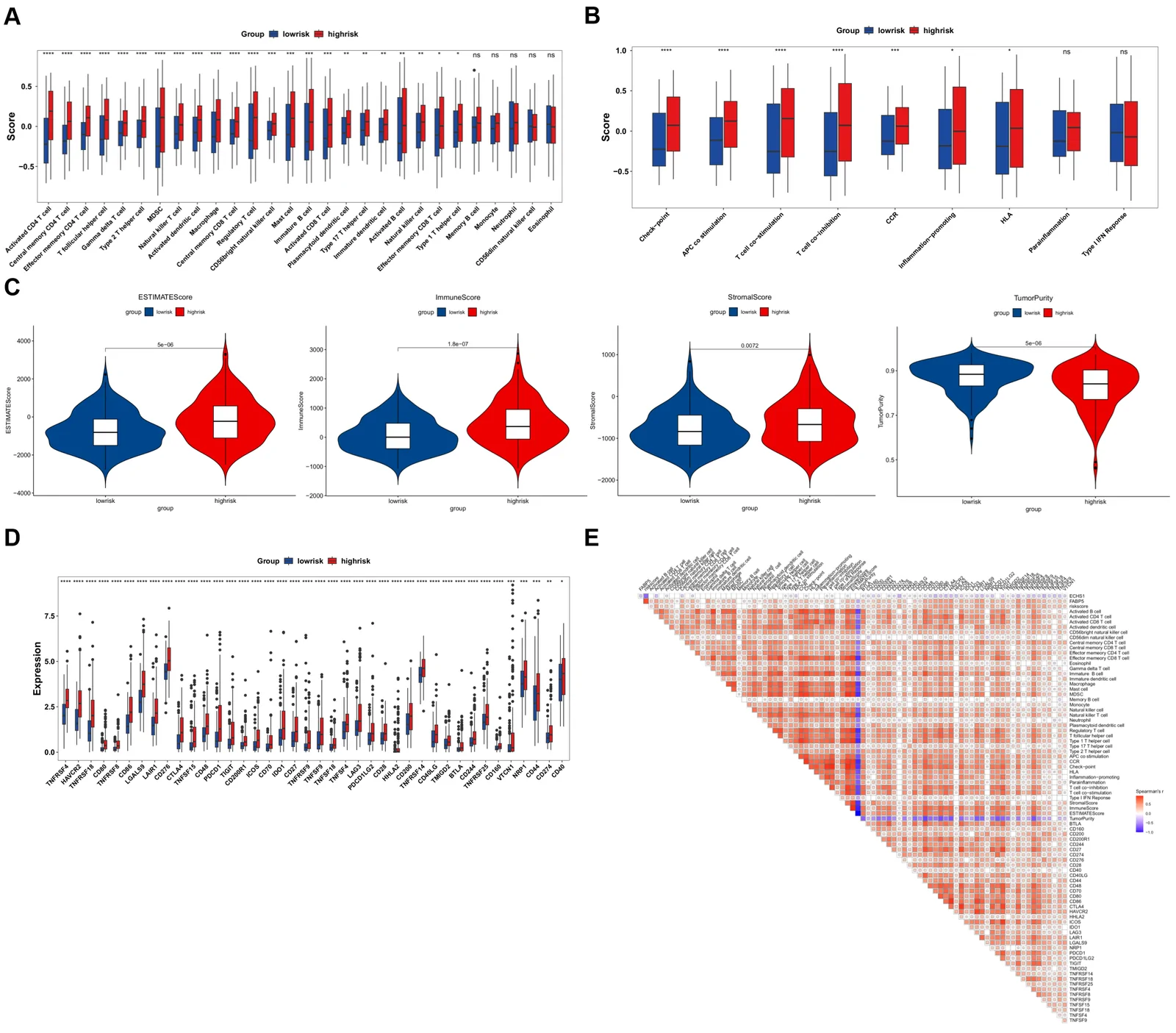
Immune landscape associated with risk score groups in HCC. Immune landscape associated with risk score groups in HCC. **(A)** Infiltration scores of 23 immune cell types were significantly higher in the HRG (p < 0.05). **(B)** Seven immune related pathways showed increased activity in the HRG. **(C)** ESTIMATE analysis: higher ESTIMATE score, immune score, and stromal score in the HRG, and lower tumor purity. **(D)** Expression of 40 immune checkpoints; TNFRSF4, HAVCR2, CD80, CTLA4, PDCD1, etc., were significantly up regulated in the HRG. **(E)** Correlation heatmap showing the strongest negative correlation between ESTIMATE score and tumor purity, and the strongest positive correlation between immune score and “check point” activity. *P < 0.05, **P < 0.01, ***P < 0.001, ****P < 0.0001.

Tumor-infiltrating immune cells strongly influence HCC progression and outcome. In the HRG, infiltration scores were significantly higher for 23 immune-cell types (p < 0.05), including activated B cells, activated CD4^+^ T cells, gamma delta T cells, immature dendritic cells, macrophages, and NK cells (**Figure 5A**). Seven immune-related pathways also had increased activity in the HRG: “APC co-stimulation”, “CCR”, “checkpoint”, “T cell co-stimulation”, “T cell co-inhibition”, “inflammation-promoting”, and “HLA” (p < 0.05, **Figure 5B**). ESTIMATE analysis showed higher ESTIMATE scores, immune scores, and stromal scores in the HRG (p < 0.01), while tumor purity was lower (p < 0.0001) (**Figure 5C**). These findings suggested that high-risk patients may have stronger immune escape-related features and a weaker tendency to respond to immunotherapy.

Checkpoint expression was also compared between the 2 risk groups. Forty immune checkpoints, including TNFRSF4, HAVCR2, CD80, LGALS9, LAIR1, CD276, CTLA4, PDCD1, TIGIT, and CD200R1, were significantly up-regulated in the HRG (p < 0.05, **Figure 5D**). Correlation analysis showed the strongest negative association between ESTIMATE score and tumor purity (cor = -1.00, p < 0.0001), whereas immune score had the strongest positive association with “check-point” activity (cor = 0.94, p < 0.0001) (**Figure 5E**; **Supplementary Table 10**). Therefore, macrophage-related lipid metabolism may be associated with abnormal immune microenvironmental alterations in HCC.

To further evaluate the external reproducibility of immune features associated with risk stratification, we performed supplementary immune analysis in the GSE14520 validation cohort. ssGSEA analysis of immune-cell infiltration and immune function showed that most immune-cell and immune-function scores did not reach statistical significance between the high- and low-risk groups, with only a few cell types showing differential trends (**Supplementary Figures 5A, B**). However, ESTIMATE analysis showed that the ESTIMATE score, ImmuneScore, and StromalScore were significantly increased in the high-risk group, whereas TumorPurity was significantly decreased (p = 0.00025, 3.0^e-06^, 0.049, and 0.00025, **Supplementary Figures 5C–F**), suggesting that the high-risk group had more prominent non-tumor cell component infiltration and lower tumor purity. Further immune checkpoint analysis showed that multiple immunomodulatory molecules, including CD86, LGALS9, TNFSF4, IDO1, CD48, CD200, NRP1, CD44, ICOSLG, CD40, LAIR1, CD160, KIR3DL1, and TNFSF14, differed significantly between the high- and low-risk groups (**Supplementary Figure 5G**). These results suggest that although specific immune-cell and immune-function scores in GSE14520 were not fully reproduced from the TCGA cohort, risk stratification-related tumor microenvironmental alterations and immune checkpoint expression differences showed a certain degree of external reproducibility.

The TIDE algorithm was further used to evaluate potential immunotherapy response tendencies. The results showed that the high-risk group had a significantly higher TIDE score, suggesting that it may have a stronger immune escape tendency and a lower possibility of benefiting from immunotherapy. Meanwhile, the cell exclusion score was also significantly higher in the high-risk group, whereas the cell dysfunction score showed no significant difference between groups (**Supplementary Figure 5H**). This result suggests that the immune escape tendency associated with the high-risk group may be reflected more in cell exclusion, enhanced stromal barriers, or changes in the immunosuppressive microenvironment. These results support, at the computational prediction level, the possibility that the high-risk group may have immune exclusion-related features, but further validation in real immunotherapy cohorts and prospective clinical samples is still required.

### 3.6 Biological mechanisms associated with risk scores in HCC

**Figure 6:**
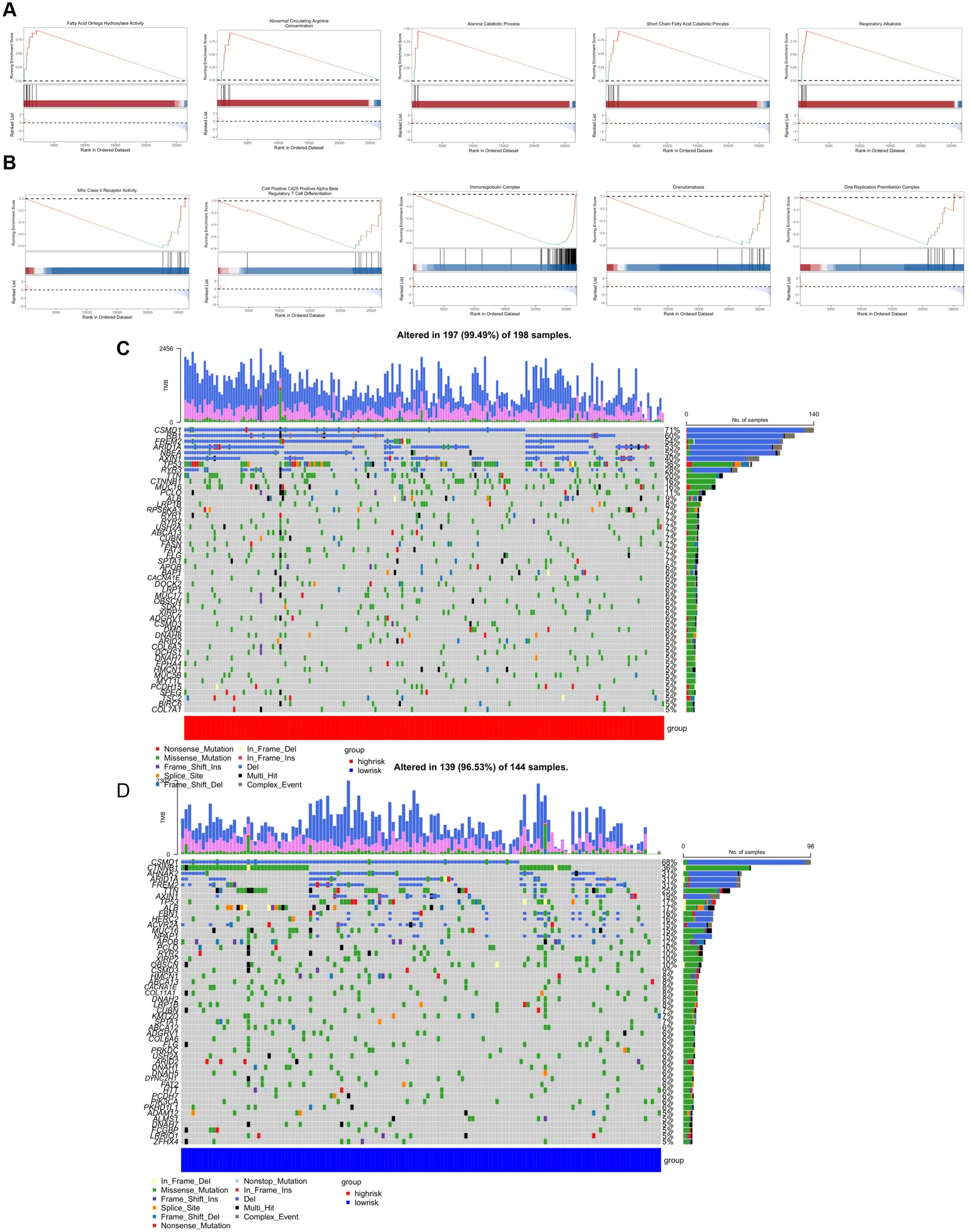
Biological mechanisms and mutation spectra associated with risk scores in HCC. **(A)** GSEA showing pathways enriched in the HRG. **(B)** GSEA showing pathways enriched in the LRG. **(C)** Top frequently mutated genes in the HRG. **(D)** Top frequently mutated genes in the LRG.

GSEA was used to explore molecular processes that may connect risk score with prognosis. In the HRG, the main enriched terms included “fatty acid omega hydroxylase activity”, “alanine catabolic process”, “abnormal circulating arginine concentration”, “short chain fatty acid catabolic process”, and “respiratory alkalosis” (p < 0.05, **Figure 6A**). In the LRG, enrichment involved “MHC class II receptor activity”, “CD4 positive CD25 positive alpha beta regulatory T cell differentiation”, “immunoglobulin complex”, “granulomatosis”, and “DNA replication preinitiation complex” (p < 0.05, **Figure 6B**; **Supplementary Table 11**). These results provided potential clues for the biological interpretation of risk stratification.

Given that FABP5 and ECHS1 may be involved in PI3K/AKT signaling, autophagy, and ferroptosis, we further calculated ssGSEA scores for related mechanistic pathways and analyzed their relationships with FABP5 and ECHS1 expression levels. The results showed that FABP5 was negatively correlated with the AKT pathway score (cor = -0.39, p < 0.001) and the autophagy pathway score (cor = -0.27, p < 0.001), whereas no significant correlation was observed between FABP5 and the PI3K or ferroptosis pathway scores. ECHS1 was positively correlated with the AKT pathway, autophagy pathway, and ferroptosis pathway scores (cor = 0.25, 0.21, and 0.21, respectively; all p < 0.001), but negatively correlated with the PI3K pathway score (cor = -0.19, p < 0.001). These results suggested that changes in FABP5 and ECHS1 expression were statistically associated with the activity of some mechanism-related pathways, providing supplementary clues for their potential involvement in HCC lipid metabolism and cell-fate regulatory processes (**Supplementary Figure 6**).

### 3.7 Mutation status associated with the Risk Score in HCC

Mutation accumulation is involved in HCC initiation and progression (40). In TCGA-HCC, deletion and missense mutations were the dominant alteration types. In the HRG, the 10 most frequently mutated genes were CSMD1, RB1, FREM2, ARID1A, NBEA, AXIN1, TP53, RYR3, TTN, and CTNNB1; CSMD1 and RB1 showed mutation frequencies of 71% and 60%, respectively (**Figure 6C**). In the LRG, the top genes were CSMD1, CTNNB1, AHNAK2, ARID1A, FREM2, TTN, AXIN1, TP53, ALB, and FBN1, with CSMD1 and CTNNB1 altered in 68% and 36% of cases (**Figure 6D**). CSMD1 mutation frequency was slightly higher in the HRG than in the LRG (71% vs. 68%). To further explore the relationship between key mutation events and the FABP5/ECHS1 risk score, TCGA-HCC patients were divided into MT and WT groups according to the mutation status of representative high-frequency mutated genes in the above mutation profile, namely CSMD1, RB1, and CTNNB1, and differences in Risk Score were compared. The results showed no significant difference in Risk Score between the CSMD1-mutant and wild-type groups (p = 0.14, **Supplementary Figure 7A**), suggesting that although CSMD1 had a high mutation frequency in both risk subgroups, its mutation status may not directly determine the FABP5/ECHS1-related risk score. In contrast, the Risk Score was significantly higher in the RB1-mutant group than in the wild-type group (p < 0.0001, **Supplementary Figure 7B**), whereas the Risk Score was significantly lower in the CTNNB1-mutant group than in the wild-type group (p = 0.0001, **Supplementary Figure 7C**). These results suggested that RB1 and CTNNB1 mutation statuses may be more closely associated with the FABP5/ECHS1-related lipid metabolism and immune-remodeling risk state.

### 3.8 Protein expression and methylation levels of FABP5 and ECHS1

**Figure 7:**
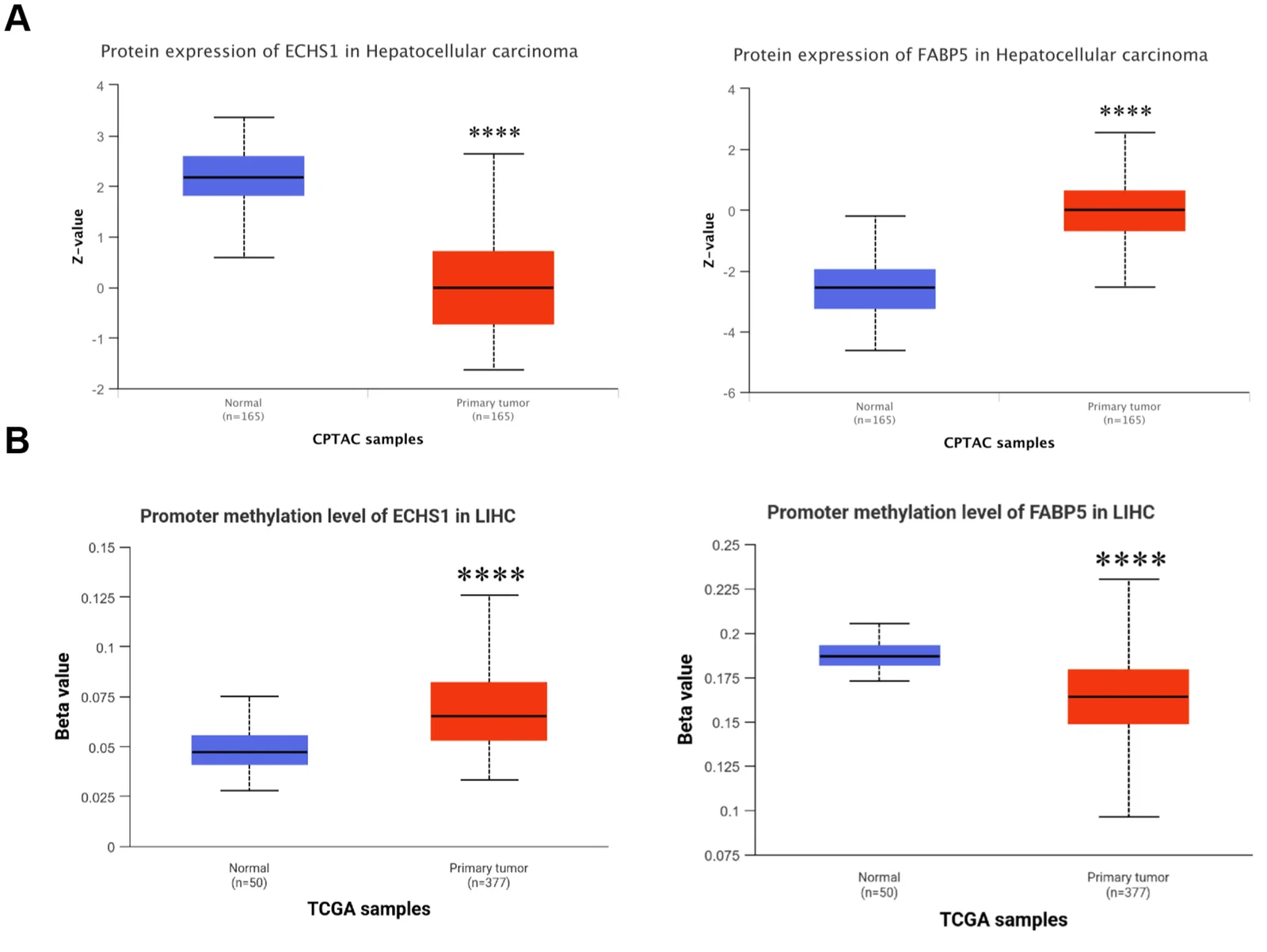
Protein expression and promoter methylation levels of FABP5 and ECHS1. **(A)** CPTAC data analysis showing increased FABP5 protein and decreased ECHS1 protein in HCC samples (p < 0.0001). **(B)** Promoter methylation analysis showing lower FABP5 promoter methylation and higher ECHS1 promoter methylation in HCC samples (p < 0.0001). ****P < 0.0001.

CPTAC data were used to examine FABP5 and ECHS1 at the protein level. FABP5 protein was elevated in HCC samples, while ECHS1 protein was reduced (p < 0.0001, **Figure 7A**). Because DNA methylation contributes to cancer initiation and progression, promoter methylation was also assessed. FABP5 promoter methylation was lower in HCC samples, whereas ECHS1 promoter methylation was higher (p < 0.0001, **Figure 7B**). These results suggested that altered FABP5/ECHS1 expression may be associated with changes at the protein level and promoter methylation status, but further experiments are needed to demonstrate their causal regulatory relationship.

### 3.9 Analysis of the status of CD8+ T cells and macrophages

**Figure 8:**
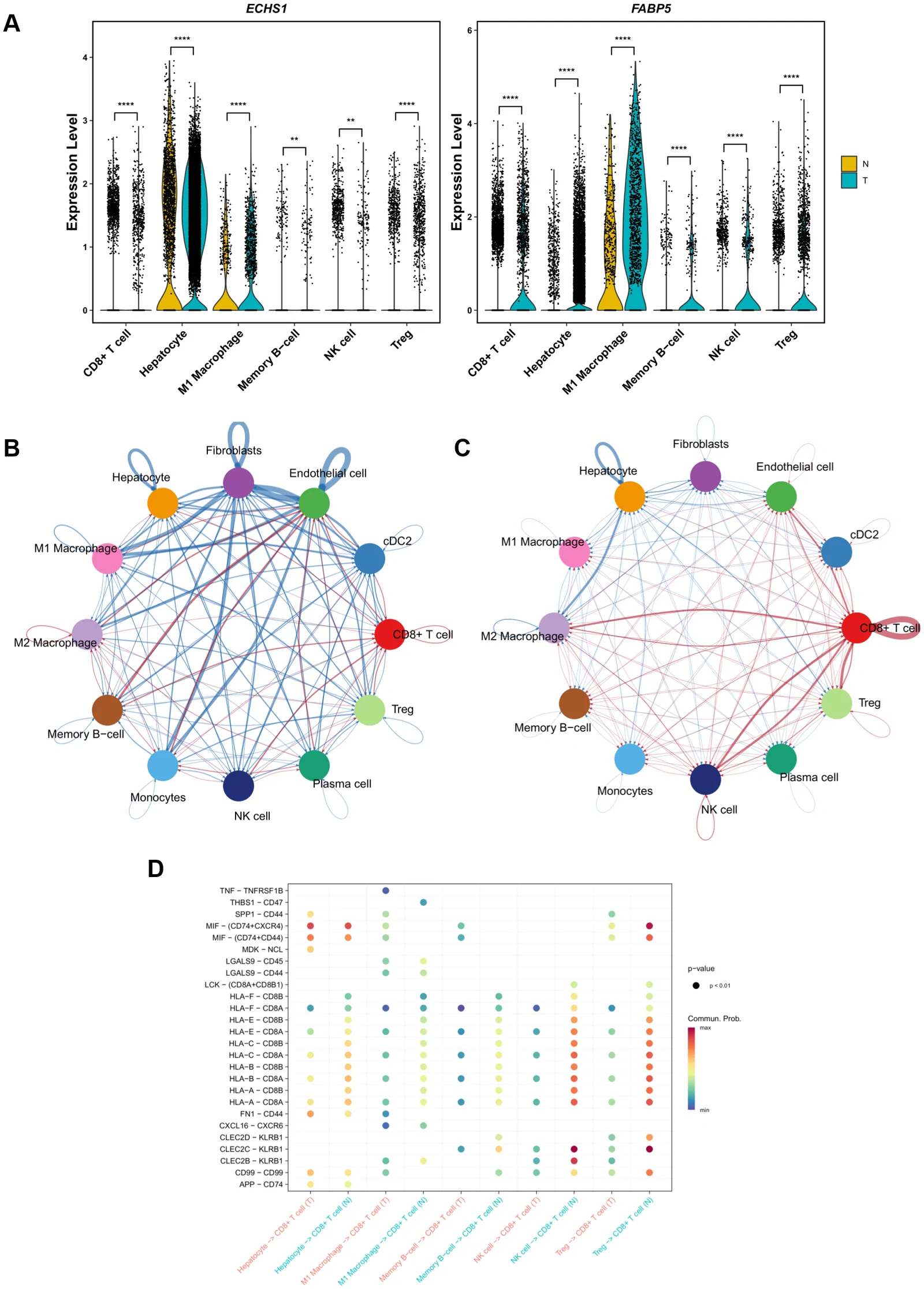
Differential expression of FABP5/ECHS1 in key cell types and cell communication network of CD8+ T cells. **(A)** Expression differences of FABP5 and ECHS1 between HCC and normal samples in CD8+ T cells, M1 macrophages, Tregs, NK cells, hepatocytes, and memory B cells (p < 0.01). **(B)** Network graph showing the number of interactions among cell types across all samples. **(C)** Network graph showing interaction strength among cell types across all samples. **(D)** Major receptor ligand pairs mediating CD8+ T cell communication with other cells. **P < 0.01, ****P < 0.0001.

The tumor microenvironment showed marked interpatient heterogeneity. FABP5 and ECHS1 were differentially expressed between HCC and normal groups in CD8^+^ T cells, M1 macrophages, Tregs, NK cells, hepatocytes, and memory B cells (p < 0.01, **Figure 8A**). Given the central regulatory role of CD8^+^ T cells in HCC and their sensitivity to tumor-microenvironmental cues, these cells were selected as key cells (41). The cell communication network summarized interaction number and strength among annotated cell types. Across all tissue samples, NK cells and CD8^+^ T cells showed strong communication (**Figures 8B, C**). CD8^+^ T-cell interactions with other cells were mainly mediated by HLA-A-CD8A, HLA-B-CD8A, and CLEC2C-KLRB1 receptor-ligand pairs (**Figure 8D**).

**Figure 9:**
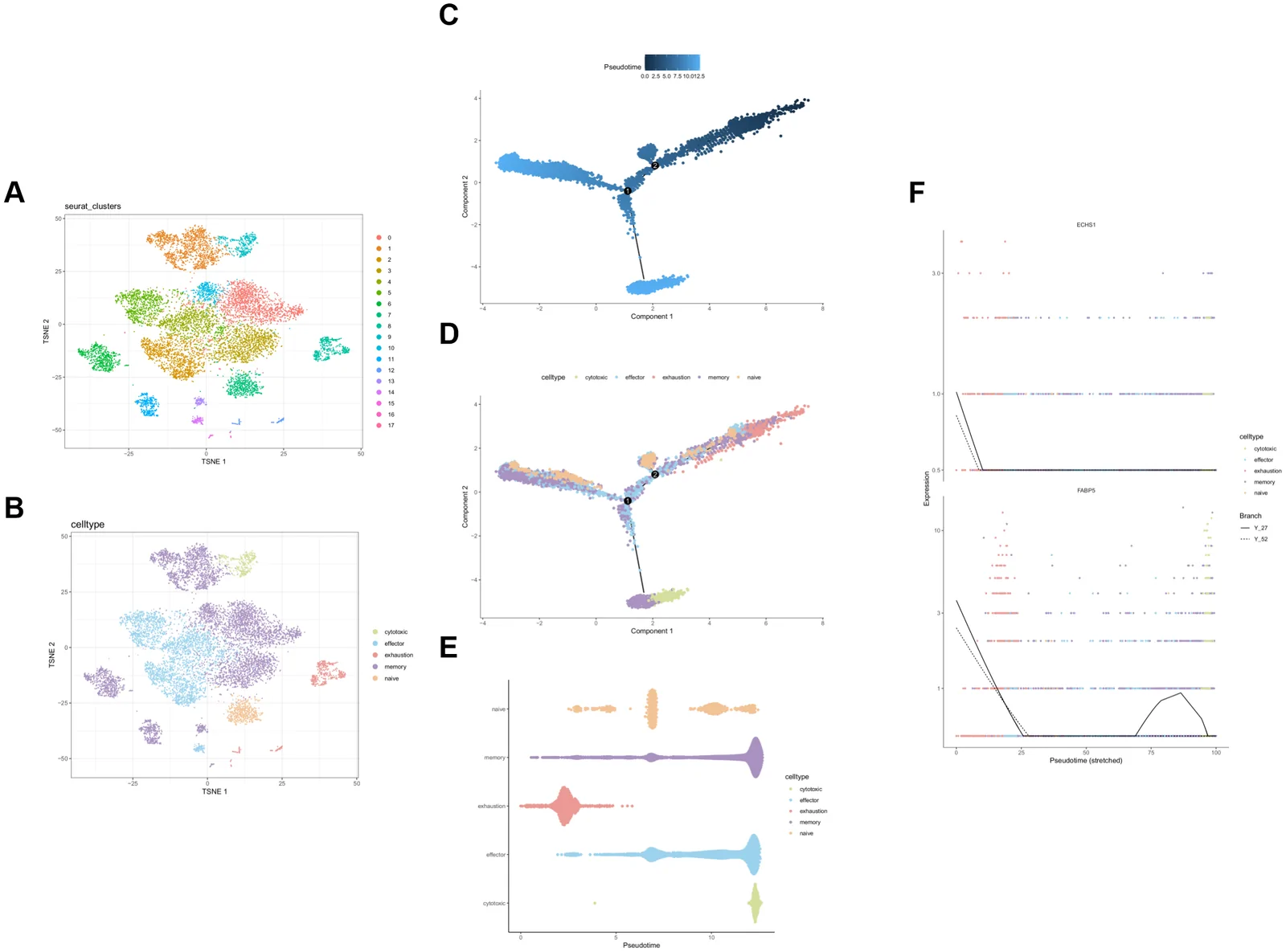
Pseudo−time differentiation trajectory analysis of CD8+ T cells. **(A)** CD8+ T cells grouped into 18 clusters. **(B)** Five subgroups: cytotoxic CD8+ T cells, effector CD8+ T cells, exhausted CD8+ T cells, memory CD8+ T cells, and naive CD8+ T cells. **(C)** Pseudo−time trajectory colored by subgroup. **(D)** Pseudo−time trajectory colored by state. **(E)** Pseudo−time trajectory colored by cluster. **(F)** Expression changes of ECHS1 and FABP5 along pseudo−time: ECHS1 initially decreased and then remained low; FABP5 showed a gradual fluctuating decline.

Pseudotime analysis was used to infer the trajectory of CD8+ T-cell state changes. The cells were grouped into 18 clusters (**Figure 9A**) and 5 subgroups: cytotoxic CD8^+^ T cells, effector CD8+ T cells, exhausted CD8^+^ T cells, memory CD8^+^ T cells, and naive CD8+ T cells (**Figure 9B**; **Supplementary Table 12**). Exhausted CD8^+^ T cells were located earlier along the trajectory, whereas memory CD8^+^ T cells appeared later (**Figures 9C–E**). Along pseudo-time, ECHS1 expression first declined and then remained low, while FABP5 showed a gradually fluctuating downward trend (**Figure 9F**). These findings suggested that changes in FABP5/ECHS1 expression may be associated with CD8+ T-cell state transitions. To further avoid overinterpretation based solely on the pseudotime trend plots, we compared the expression differences of FABP5 and ECHS1 between the HCC and normal groups across different CD8+ T-cell subpopulations. The results showed that FABP5 differed significantly between groups in effector CD8+ T cells and cytotoxic CD8+ T cells, whereas its expression was low or not clearly detected in exhausted CD8+ T cells from the normal control group. ECHS1 differed significantly between groups in naive CD8+ T cells, memory CD8+ T cells, and effector CD8+ T cells, whereas its expression was low or not clearly detected in exhausted CD8+ T cells from the normal control group (**Supplementary Figure 8**). These results suggest that the expression differences of FABP5 and ECHS1 in CD8+ T cells show a degree of subset specificity; however, these observations are expression-based associations and cannot demonstrate that the two genes directly exert causal effects on CD8+ T-cell exhaustion, memory differentiation, or other state transitions.

We further performed a supplementary pseudotime analysis of M1 and M2 macrophages. After M1 and M2 macrophages were merged and reclustered, 18 macrophage clusters were obtained (**Supplementary Figure 9A**), and their M1/M2 cell-type distribution was visualized (**Supplementary Figure 9B**). Monocle pseudotime analysis showed that macrophages exhibited a continuous state distribution along the pseudotime trajectory, with M1 and M2 macrophages showing certain distribution differences at different pseudotime stages (**Supplementary Figures 9C–E**). Further subpopulation expression analysis showed that FABP5 and ECHS1 differed significantly between the HCC and normal groups in both M1 and M2 macrophages (**Supplementary Figure 9F**). These results further supported an association between FABP5/ECHS1 and macrophage state changes and also indicated that selecting CD8+ T cells as key cells for communication and pseudotime analyses did not exclude the important role of macrophages in MLMG-related immunometabolic remodeling.

### 3.10 Expression confirmation of FABP5 and ECHS1

**Figure 10:**
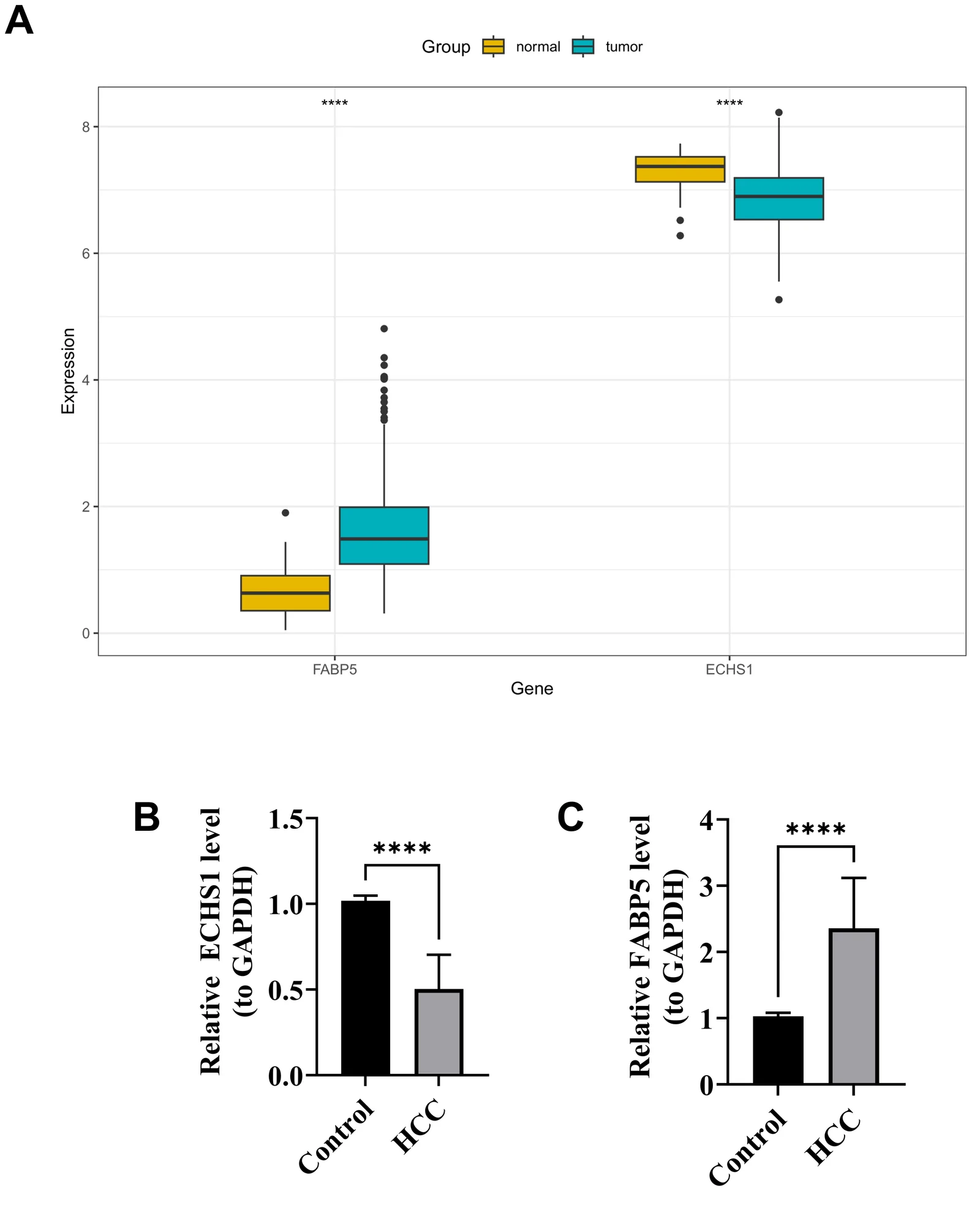
Validation of FABP5 and ECHS1 expression in HCC. **(A)** TCGA-HCC data analysis showing significantly higher FABP5 expression and significantly lower ECHS1 expression in HCC patients (p < 0.0001). **(B)** RT-qPCR validation of FABP5 mRNA up-regulation in HCC patients (p < 0.0001). **(C)** RT-qPCR validation of ECHS1 mRNA down-regulation in HCC patients (p < 0.001). ****P < 0.0001.

TCGA-HCC analysis linked FABP5 and ECHS1 to HCC. FABP5 expression was significantly higher in HCC patients than in normal controls (p < 0.0001), while ECHS1 expression was significantly lower (p < 0.0001) (**Figure 10A**). The RT-qPCR results were preliminarily consistent with the bioinformatics findings, showing downregulated ECHS1 mRNA (p < 0.0001, **Figure 10B**) and upregulated FABP5 mRNA (p < 0.001, **Figure 10C**) in patients with HCC. These findings suggested that prognostic MLMGs may be related to HCC-associated molecular alterations.

## 4 Discussion

This study integrated bulk transcriptomic and single-cell RNA sequencing data and identified 20 macrophage-associated DE-MLMGs in HCC. LASSO regression reduced these genes to a 2-gene prognostic model in which FABP5 contributed risk and ECHS1 contributed protection. The risk score was calculated as risk score = (-0.1982) * ECHS1 expression + (0.2526) * FABP5 expression. This signature separated patients into high- and low-risk groups in TCGA-HCC and GSE14520. In the training cohort, high-risk patients had poorer OS (p = 0.0059), and 1-, 2-, and 3-year OS AUC values were 0.65, 0.60, and 0.60, indicating moderate prediction. RT-qPCR preliminarily supported the computational results, with higher FABP5 (p < 0.0001) and lower ECHS1 (p < 0.001) in HCC tissues, consistent with their proposed oncogenic and tumor-suppressive roles.

It should be noted that the predictive performance of the FABP5/ECHS1 model should be interpreted in the broader context of HCC prognostic modeling. HCC has substantial molecular and clinical heterogeneity, and differences in etiological background, treatment modality, tumor stage, and follow-up duration may affect the predictive stability of transcriptomic models (42). Similar moderate AUCs are also relatively common in previous molecular prognostic models for HCC. For example, in the external GSE14520 validation cohort, the immune-related HCC prognostic model constructed by Wang et al. had 1-, 3-, and 5-year AUCs of 0.60, 0.61, and 0.60, respectively (43). The two-exosomal-gene model constructed by Zhu et al. also showed moderate AUCs in two validation cohorts; in one validation cohort, the AUCs at 0.5, 1, 3, and 4 years were 0.66, 0.61, 0.62, and 0.59, respectively (44). Therefore, the present model may be more suitable as a supplementary prognostic stratification tool for traditional clinicopathological staging rather than as a replacement for existing staging systems or a tool to independently determine treatment strategies. Compared with some multigene models, this model contains only two genes, FABP5 and ECHS1, and is structurally simpler. Because it was derived from the intersection of macrophage-related genes, lipid metabolism-related genes, and HCC differentially expressed genes, it more specifically reflects the macrophage-lipid metabolism reprogramming axis in HCC. Further analyses show that the Risk Score is not only associated with survival differences, but also linked to increased immune infiltration, upregulated immune checkpoints, immune escape-related features, intercellular communication patterns, and CD8+ T-cell pseudotime changes, suggesting that the value of this model is not limited to risk scoring but may also provide potential clues for understanding immunometabolic remodeling in HCC. Larger multicenter prospective cohorts are needed to further validate its clinical translational value.

FABP5 may support HCC progression by linking fatty acid handling to HIF-1α and PI3K/Akt/mTOR signaling, thereby promoting proliferation and lipid metabolic remodeling (45, 46). Recent reports provide additional mechanisms. In obesity-associated HCC, FABP5 has been associated with ferroptosis suppression and an immunosuppressive microenvironment, possibly through TAM polarization toward a pro-tumorigenic phenotype (47). Its macrophage relevance is supported by detection in profibrogenic macrophage subsets in human liver fibrosis (48) and by possible involvement in hypoxia-induced exosomal miR-4488/RTN3 signaling, which may promote M2-like polarization and fatty-acid-oxidation-dependent reprogramming (49). FABP5 may maintain CD8+ T-cell activity but may also enhance the suppressive effects of Tregs and TAMs, thereby favoring HCC progression (50, 51). It has also been linked to liver cancer stemness through ACSL4-mediated fatty acid β-oxidation (52). ECHS1 is a mitochondrial β-oxidation enzyme with potential tumor-suppressive activity in HCC (53, 54). Reduced ECHS1 is associated with ATP depletion, AMPK activation, autophagy induction, and reduced proliferation (55). ECHS1 knockdown can enhance HCC-cell proliferation, suppress apoptosis, and activate Akt signaling, whereas overexpression has opposite effects (53). Lower ECHS1 also predicts worse prognosis in chronic hepatitis B with non-alcoholic fatty liver disease (54), and in HBV-related HCC it may interact with HBsAg to induce mitochondrial apoptosis (56). The inverse FABP5/ECHS1 pattern therefore suggests opposing influences on lipid metabolism, ferroptosis, stemness, and immune regulation.

The 20 DE-MLMGs were enriched in fatty acid metabolism, steroid metabolism, peroxisomal processes, PPAR signaling, fatty acid metabolism, and primary bile acid biosynthesis (57, 58). PPAR signaling is a key interface between lipid metabolism and immune regulation (59, 60). In HCC, FABP5 and related fatty acid binding proteins may deliver ligands to activate PPARγ, increasing immunosuppressive molecules, favoring M2-like TAM polarization, and contributing to immune checkpoint inhibitor (ICI) resistance (60, 61). PPARα appears protective by promoting lipophagy and restraining NF-κB and NLRP3 inflammasome activity; loss of PPARα function can accelerate HCC progression (59, 62). Several upstream factors modulate this pathway. CES1 generates polyunsaturated fatty acids (PUFAs), natural PPARα/γ ligands; CES1 inhibition lowers PUFA availability, reduces PPARα/γ-driven SCD expression, impairs mitochondria, and increases cisplatin sensitivity (63). CRSP8 promotes CRM1-mediated nuclear export of PPARα through RAN, which lowers nuclear PPARα, suppresses lipophagy and fatty acid oxidation (FAO), and promotes lipid-droplet accumulation and HCC progression (64). Conversely, the SIRT1/PGC-1α/PPARα axis enhances FAO and alleviates steatohepatitis, potentially limiting NASH-to-HCC transition (65, 66). SREBP1-dependent *de novo* lipogenesis also participates in HCC lipid remodeling; MARCH8 degrades SREBP1 to suppress HCC, whereas TRIM45 accelerates NASH-to-HCC transition through the FABP5-PPARγ axis (67–69). Butyrate may potentiate anti-PD-1 therapy by inducing GPX4 ubiquitination, degradation, and ferroptosis (70). AF6 knockout enhances bile acid production and butyrate levels, recruiting dendritic cells through CXCL14 to activate CD8+ T-cell anti-tumor immunity (71). SCFA catabolism remains context dependent, as butyrate may enhance NK-cell chemotaxis and CD8+ T-cell cytotoxicity but can have tumor-promoting effects in some settings (70, 72). The PPI network placed ALB, APOA1, GC, FABP1, FABP4, FABP5, PLIN2, ACOX2, and SCP2 centrally, suggesting that this lipid metabolism-PPAR network may offer targets for chemoresistance and immunotherapy resistance.

Despite the higher immune infiltration scores observed in the HRG, increased immune cell numbers do not necessarily indicate effective antitumor immune responses. According to the “cold/hot tumor” theory, some tumors with substantial immune cell infiltration may exhibit a functionally hot but effector-restricted tumor microenvironment or an immune-excluded state, due to co-upregulation of immune checkpoints, accumulation of immunosuppressive myeloid cells, impaired antigen presentation, and stromal barrier formation (73, 74). In our study, the TCGA cohort HRG showed elevated infiltration scores for multiple immune cell types, accompanied by increased immune and stromal scores, decreased tumor purity, and upregulation of various immune checkpoint molecules; similar ESTIMATE-related trends were observed in the GSE14520 validation cohort. These results suggest that although the high-risk group has a richer immune and stromal component, its antitumor efficacy may be suppressed. Specifically, tumor-associated macrophages (TAMs) are the predominant immune cells in the HCC microenvironment and can contribute to immune evasion by secreting immunosuppressive factors, promoting T-cell suppression, and supporting tumor progression (7); immature or dysfunctional dendritic cells may impair antigen presentation and T-cell priming, thus limiting the translation of immune infiltration into effective cytotoxic responses (75); and sustained antigen exposure and inflammatory stimuli can induce continuous expression of multiple inhibitory receptors on T cells, progressively restricting their effector functions (76). In addition, the elevated stromal score in HRG suggests increased CAF/ECM components (77), and CAFs and ECM can form physical and signaling barriers that restrict CD8+ T-cell entry into tumor nests or impair their killing functions (78). Notably, TIDE analysis revealed that HRG had elevated TIDE scores and cell exclusion scores, whereas cell dysfunction scores showed no significant difference, further indicating that immune evasion in the high-risk group may be more attributed to cell exclusion, stromal barriers, and myeloid immunosuppression rather than a generalized increase in overall cellular dysfunction. Therefore, HRG may represent an immunosuppressive/immune-excluded state with abundant immune infiltration but limited effector functionality, which could partially explain the paradoxical phenomenon of higher immune infiltration yet poorer prognosis.

At the mutation level, CSMD1 showed relatively high mutation frequencies in both the HRG and LRG (71% vs. 68%). Although previous studies regard CSMD1 as a potential tumor suppressor in HCC (79–82), the risk score did not differ significantly between patients with mutant and wild-type CSMD1 in the present study (p = 0.14), which does not support a significant association between CSMD1 mutation status and the FABP5/ECHS1 risk score. By contrast, the risk score was significantly higher in patients with RB1 mutation (p = 7.2e-07), whereas it was significantly lower in patients with CTNNB1 mutation (p = 1.0e-04), suggesting that the statistical associations of RB1 and CTNNB1 mutations with this risk score may deserve greater attention. However, these findings are still cross-sectional associations and cannot indicate a direct causal relationship between driver mutations and the FABP5/ECHS1-related immunometabolic state; further validation is needed.

Cell communication analysis showed the strongest crosstalk between NK cells and CD8+ T cells. CD8+ T cells mainly communicated with other cell types through HLA-A/CD8A, HLA-B/CD8A, and CLEC2C/KLRB1 receptor-ligand pairs (83, 84). Pseudotime reconstruction separated CD8+ T cells into five subsets, with exhausted cells positioned early and memory cells late. FABP5 progressively declined along the trajectory, whereas ECHS1 first decreased and then stayed low. Because FABP5 supports fatty acid oxidation and anti-apoptotic capacity in exhausted T cells (Tex) (42), its decline may reflect inadequate metabolic adaptation. A CD8+ T-cell-related signature from Wherry et al. also included FABP5 and was associated with immune escape in the high-risk group (76). ECHS1 is essential for mitochondrial β-oxidation; in gastric cancer, its downregulation suppresses inflammatory cytokines and microenvironmental heterogeneity (85). In this study, persistently low ECHS1 levels may be related to AMPK-mediated autophagy, altered mitochondrial function, and CD8+ T-cell survival states. Kuwano et al. showed that CD8+ TIL counts combined with serum CRP predict immunotherapy efficacy in HCC, underscoring that abundance does not equal function (86). Therefore, the pseudotime findings of this study provide single-cell-level support for a potential link between FABP5/ECHS1 and the metabolic and functional states of CD8+ T cells and may help explain the phenomenon of increased immune infiltration but restricted antitumor effects in high-risk HCC. It should be noted that this analysis reflects associations between expression changes and pseudotime states; it cannot determine whether FABP5 or ECHS1 directly drives CD8+ T-cell exhaustion, memory differentiation, or immune escape, and their specific roles require further functional validation.

The FABP5/ECHS1 model may aid HCC risk stratification and individualized treatment design. Other lipid metabolism-based signatures have also predicted HCC prognosis and related to immunosuppressive microenvironments and ICB response (87). PPARγ inhibition with T0070907 can overcome ICB resistance in HCC by increasing cytolytic activity, reducing T-cell exhaustion, and decreasing MDSC infiltration (60). Butyrate may also enhance ICI efficacy through ferroptosis induction and immune-microenvironment modulation (70). Because the HRG displayed a “functional hot tumor” phenotype, NKG2A-, TIGIT-, or CAF/ECM-directed combinations should be evaluated in biomarker-selected cohorts rather than assuming that anti-PD-1 monotherapy is adequate. The model may help select such patients and inform personalized immunotherapy trials. FABP5 inhibition or ECHS1 restoration may also be therapeutically relevant and should be evaluated in future combined-targeting strategies (88).

This study still has certain limitations. First, although survival validation is performed in GSE14520, and immune microenvironment features and TIDE predictions are further analyzed, independent validation in immune checkpoint inhibitor-treated HCC cohorts, real-world samples, and prospective cohorts is currently lacking. Therefore, inferences regarding immune evasion and treatment response still need to be interpreted with caution. Second, the mechanistic analysis in this study is mainly based on transcriptomic data, single-cell data, pathway scores, and previous literature; it cannot yet directly demonstrate that FABP5 and ECHS1 causally regulate PI3K/AKT, PPAR, autophagy, ferroptosis, macrophage polarization, or CD8+ T cell exhaustion. Future studies are still needed to use FABP5/ECHS1 overexpression or knockdown, TAM–HCC cell co-culture, macrophage–CD8+ T cell co-culture, T cell functional assays, and *in vivo* models to further validate their direct biological functions. Third, although RT-qPCR validation is performed with a sample size of 20 pairs of HCC tissues and matched control tissues, which enhances the reliability of the expression validation results, this sample size remains insufficient to fully reflect the significant clinical and etiological heterogeneity of HCC and to allow robust stratified analyses. Future studies should be validated in larger independent clinical cohorts with more complete clinical characteristics, and stratified analyses should be conducted according to different etiological backgrounds, such as HBV-, HCV-, NASH-, and alcohol-related HCC. Fourth, the relationship between FABP5 and ECHS1 promoter methylation and their expression changes currently shows only a correlation, and it cannot yet be proven that epigenetic modifications directly and causally regulate their mRNA or protein expression. Subsequent studies need to be validated using demethylating agent treatment, methylation-specific PCR, promoter reporter gene assays, or targeted epigenetic editing. Finally, some clinical information in public databases is missing, such as AFP levels, specific treatment regimens, and viral infection status, which limits more comprehensive confounder adjustment and subgroup analyses. Future research should integrate multi-omics data, immunotherapy outcomes, and functional experimental validation in multicenter cohorts with more complete clinical information to further elucidate the potential application value of the FABP5/ECHS1-related MLMG model in HCC prognosis assessment and immune-metabolism therapeutic research.

## Data Availability

The datasets presented in this study can be found in online repositories. The names of the repository/repositories and accession number(s) can be found in the article/**Supplementary Material**.
